# A novel non genomic glucocorticoid signaling mediated by a membrane palmitoylated glucocorticoid receptor cross talks with GnRH in gonadotrope cells

**DOI:** 10.1038/s41598-017-01777-2

**Published:** 2017-05-08

**Authors:** Mohsen Ayrout, Violaine Simon, Valérie Bernard, Nadine Binart, Joëlle Cohen-Tannoudji, Marc Lombès, Stéphanie Chauvin

**Affiliations:** 1 INSERM UMR_S1185, Fac Med Paris Sud, Université Paris-Saclay, F-94276 Le Kremlin Bicêtre, France; 2 Sorbonne Paris Cité, Université Paris-Diderot, CNRS, INSERM, Biologie Fonctionnelle et Adaptative UMR 8251, Physiologie de l’axe gonadotrope, U1133 Paris, France

## Abstract

Glucocorticoid hormones (GC) are the main stress mediators associated with reproductive disorders. GC exert their effects through activation of the glucocorticoid receptor (GR) principally acting as a transcription factor. Beside well-established GR-mediated genomic actions, several lines of evidence suggest a role for rapid membrane-initiated GC signaling in gonadotrope cells triggered by a membrane-associated GR. Herein, we demonstrate the existence of a specific membrane-initiated GC signaling in LβT2 gonadotrope cells involving two related phosphoproteins: Ca^2+^/Calmodulin-dependent protein kinase II (CaMKII) and synapsin-I. Within 5 min, LβT2 cells treated with stress range of 10^−7^ M Corticosterone or a membrane impermeable-GC, BSA-conjugated corticosterone, exhibited a 2-fold increase in levels of phospho-CaMKII and phospho-synapsin-I. Biochemical approaches revealed that this rapid signaling is promoted by a palmitoylated GR. Importantly, GC significantly alter GnRH-induced CaMKII phosphorylation, consistent with a novel cross-talk between the GnRH receptor and GC. This negative effect of GC on GnRH signaling was further observed on LH release by mouse pituitary explants. Altogether, our work provides new findings in GC field by bringing novel understanding on how GR integrates plasma membrane, allowing GC membrane-initiated signaling that differs in presence of GnRH to disrupt GnRH-dependent signaling and LH secretion.

## Introduction

Glucocorticoid hormones (GC) are the main mediators of stress and their increased secretion leads to a large variety of acute and long-term responses. Physical and emotional stressors have been reported to perturb female menstrual cycles^1, 2^, to alter the onset of puberty^3^, to be associated with a longer time-to-pregnancy and to increase the risk of infertility^4^. Psychosocial stress has been extensively studied in ewes^5^. It is now established that GC act at different levels of the reproductive axis: at the hypothalamus by reducing Gonadotropin-releasing-hormone (GnRH) pulse frequency^6^ and at the pituitary by rapidly decreasing responsiveness to GnRH to suppress pulsatile gonadotropins expression and secretion, notably LH^7^. GC responses are mediated by the glucocorticoid receptor (GR), a member of the nuclear receptor superfamily, through two separate but inter-related mechanisms of actions^8^. After ligand binding, GC/GR complexes translocate into the nucleus to regulate target genes expression^9^. Several studies described a direct action of GC in pituitary gonadotrope cells to regulate gonadotropins and GnRH receptor (GnRH-R) expression^10^. In addition, signaling pathways mediating rapid non-genomic GC actions have been demonstrated in a number of studies; they involve various cytosolic proteins^11^ *e*.*g* Phospholipase C^12^, Mitogen-Activated Protein Kinases (MAPK)^13, 14^, Phosphatidylinositol 3-kinase^15^, Src kinase^16, 17^ or intracellular Ca^2+^ signaling^18^.

GC-induced rapid signaling has never been directly studied in gonadotrope cells yet, different arguments suggest their involvement in the rapid blunting of GnRH responsiveness during stress^7^. We speculate that GC could modulate signaling mechanisms downstream of the GnRH-R leading to a reduction of gonadotropin release. Such GC-induced rapid signaling should involve the activation of a putative membrane associated GR (MbGR). The existence of such a MbGR has been convincingly demonstrated in monocytes and B lymphocytes^19^, keratinocytes^20^, hippocampal neurons^21^ or in several cell lines^16, 22, 23^. Studies using classical GR antibodies^24, 25^ or using RNA interference-mediated GR reduction^26^ revealed that intracellular GR and MbGR may originate from the same gene. Membrane targeting of a steroid receptor has been already described for the estrogen receptor (ER) which undergoes membrane translocation after caveolin-1 association^27^ and protein palmitoylation^28^. Palmitoylation is a posttranslational process by which palmitate, a C16 fatty acid, is covalently linked to an internal cysteine *via* a thioester bond. As for ER, GR has been found associated with lipid rafts^29^ and caveolin-1^14, 30, 31^. However, contrary to ER, Vernocchi *et al*., proposed that the membrane localization of GR is not totally dependent on caveolin-1^32^. Posttranslational modifications that influence GR membrane localization^33^ have not been described yet.

In the present study, we used a murine gonadotrope cell line (LβT2) expressing GR and prone to release LH upon GnRH treatment, as well as mouse pituitary explants to better understand whether and how GC might rapidly interfere with GnRH signaling to prevent LH release during stress. Our data provide evidence, using a membrane-impermeable corticosterone conjugate, Cort-BSA (bovine serum albumin), and an inhibitor of protein palmitoylation (2-Br) that GC induce a novel rapid signaling in LβT2 cells through the activation of a palmitoylated membrane GR. We also demonstrate that GC interfere with GnRH induced Ca^2+^/Calmodulin-dependent kinase 2 (CaMKII) phosphorylation in LβT2 cells, ultimately decreasing LH release by pituitary explants.

## Results

### Specific membrane-initiated GC signaling in gonadotrope cells: role of CaMKII and synapsin-I

**Figure 1:**
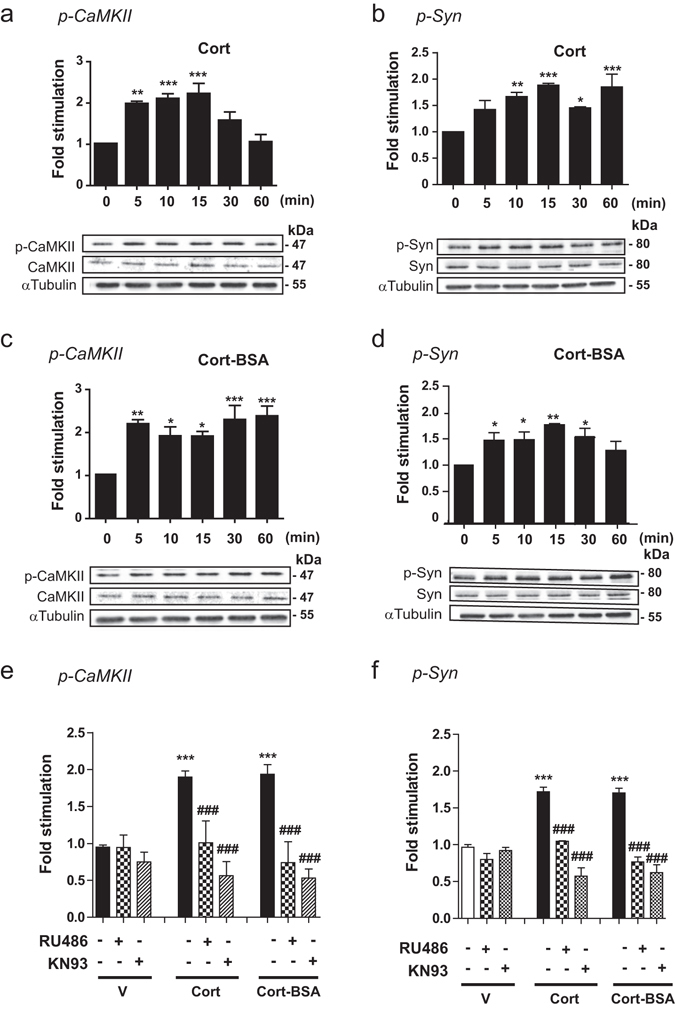
GC rapidly stimulate CaMKII and synapsin-I phosphorylation in LβT2 cells. (**a**,**b**) LβT2 cells were treated with Cort (10^−7^ M) for 0 to 60 min. Cell lysates were subjected to western blotting (WB) with anti-CaMKII (**a**), anti-p-CaMKII (**a**), anti-synapsin (**b**), anti-p-synapsin (**b**) and anti-α-tubulin antibodies (**a**,**b**). Cropped blots are shown in the figure and are representative of 4 independent experiments. Results are expressed as means ± SEM fold stimulation when compared to time 0. One-way ANOVA with Dunnett’s posttest, *p < 0.05, **p < 0.01, and ***p < 0.001. (**c**,**d**) LβT2 were treated for 0 to 60 min with Cort-BSA (10^−7^ M). Cropped immunoblots are shown in the figure and are representative of 4 independent experiments. Results are expressed as means ± SEM fold stimulation when compared to time 0, one-way ANOVA with Dunnett’s posttest, *p < 0.05, **p < 0.01. (**e**,**f**) Phosphorylated CaMKII (**e**) and Syn (**f**) were significantly increased after 5 min treatment with either Cort or Cort-BSA. These events were abolished by co-incubation with RU486 (10^−5^ M) or KN93 (10^−5^ M). V: Vehicle. Statistical significance was assessed by one-way ANOVA with Newman-Keuls Multiple Comparison Test, n ≥ 3, ***p < 0.001 when compared to Vehicle, ^###^ *p* < 0.001 within the same steroid treatment (Cort or Cort-BSA). Full length WBs are presented in Supplementary Fig. S4a–c. p-CaMKII: phospho-CaMKII; p-Syn: phospho-synaspin I.

In ewes, GC have been described to reduce the LH response of pulse-like delivery of GnRH by 50% within 30 min, indicating rapid action of GC on pituitary^7^. To examine a potential relationship between rapid GC-induced signaling and LH release reduction, we investigated whether specific rapid GC signaling pathways were activated in gonadotrope cells. LβT2 cells were treated with a stress range of 10^−7^ M corticosterone (Cort) for up to 60 min and the level of phosphorylation of CaMKII (p-CaMKII) as well as one of its target, synapsin-I (Syn) were evaluated by Western blotting; signals were normalized to the level of total proteins and α-tubulin (Fig. 1a,b). Figure 1a shows that Cort induced a rapid and transient phosphorylation of CaMKII in LβT2 cells with a maximum (about 2.3-fold) observed after 15 min of treatment which then returned to basal value. Cort also stimulated the level of phospho-Syn (p-Syn) within 10 min (Fig. 1b) and such an effect was maintained up to 60 min of treatment. Cort treatment did not modify total levels of CaMKII and Syn. To bring support for a membrane GR-initiated effect, we used BSA-conjugated corticosterone (Cort-BSA), a membrane impermeable GC that allows discriminating specific activities of the MbGR from those of the intracellular GR. The ability of Cort-BSA to promote genomic effects was tested on the expression of a GC-induced gene Serum and Glucocorticoid regulated Kinase 1 (*Sgk-1*). As shown in Supplementary Fig. S1, Cort-BSA did not induce expression of *Sgk-1* after 1 or 4 h of treatment, when compared to vehicle, in contrast to the expected effects of dexamethasone (Dex, a GR agonist) or Cort. These data confirm that Cort-BSA effects could only be attributed to non-genomic mechanisms in LβT2 cells. Exposure of LβT2 cells to Cort-BSA (10^−7^ M) for 5 min induced the same level (about 2-fold) of CaMKII phosphorylation as the one induced by Cort, but phosphorylation remained stable up to 60 min of treatment (Fig. 1c). A similar phosphorylation pattern was observed for p-Syn following Cort-BSA and Cort exposure, except an earlier return to basal level after Cort-BSA treatment (Fig. 1d). Total levels of CaMKII and Syn were unchanged after Cort-BSA treatment. Interestingly, Dex did not stimulate MAPK (Supplementary Fig. S2a) nor Src signaling pathways (Supplementary Fig. S2b) in contrast to their reported effect in most cellular models^11^ studied so far. The specificity of Cort/Cort-BSA rapid signaling was assessed using a GR antagonist (RU486) or a CaMKII inhibitor (KN93) (Fig. 1e). Co-incubation of Cort or Cort-BSA with these inhibitors prevented CaMKII phosphorylation measured after 5 min of treatment in LβT2 cells, without significantly affecting basal p-CaMKII. Interestingly, both inhibitors also affected the level of GC-induced p-Syn observed after 5 min of treatment without disturbing basal level of p-Syn (Fig. 1f). These data provide evidence that Cort or Cort-BSA rapidly promotes the phosphorylation of CaMKII and Syn in gonadotrope cells. This phosphorylation sequence therefore constitutes a new GC membrane-initiated signaling dependent on GR.

**Figure 2:**
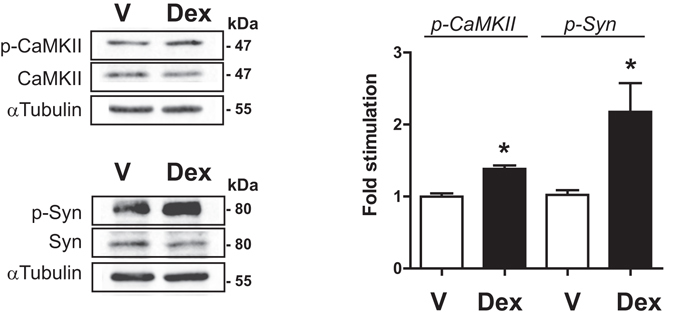
Dexamethasone stimulates phosphorylation of CaMKII and Syn in mouse pituitary explants. Explanted pituitaries were treated with Dex (10^−7^ M) or not (Vehicle, V) for 30 min. Lysates were subjected to immunoblotting with anti-p-CaMKII, anti-CaMKII, anti-Syn, anti-p-Syn and anti-α-tubulin antibodies. Dex induced significant increase in the phosphorylation state of CaMKII and Synapsin-I. Cropped blots (full length WBs are presented in Supplementary Fig. S4d) are shown in the figure and are representative image of 3 independent experiments. Results are expressed as means ± SEM fold stimulation when compared to vehicle. Statistical significance was assessed by One way ANOVA, Dunnett’s posttest, n = 3, *p ≤ 0.05. p-CaMKII: phospho-CaMKII; p-Syn: phospho-synaspin-I.

We next examined GC effects in a more physiological context. To this aim, mouse pituitary explants were treated for 30 min with the GR agonist Dex. We found a significant GC-induced increase in levels of p-CaMKII (about 1.4-fold) as well as in p-Syn (about 2.2-fold) (Fig. 2). Dex treatment did not alter total CaMKII or total Syn contents. This effect was prevented by co-treatment with RU486 (not shown). CaMKII and Syn therefore constitute new key elements of GC membrane-initiated signaling in pituitary.

### A palmitoylated membrane GR promotes CaMKII phosphorylation in LβT2 cells

**Figure 3:**
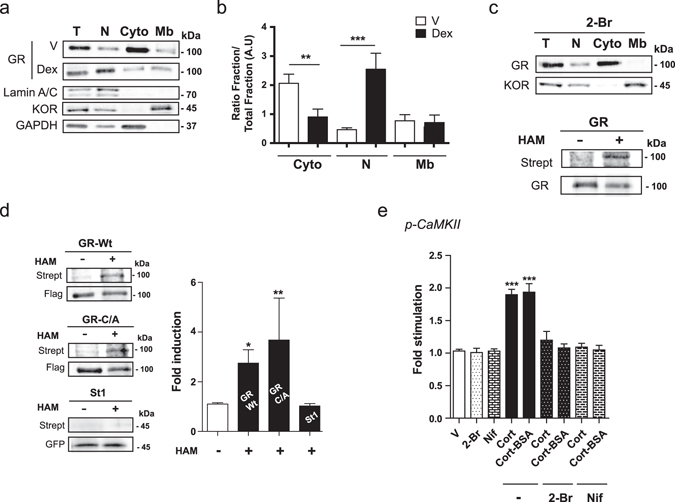
A palmitoylated membrane GR is involved in GC-induced rapid CaMKII phosphorylation in LβT2 cells. (**a**) LβT2 cells were treated or not (V) with Dex before subcellular fractionation to detect GR and specific markers by WB; lamin A/C, κ-opioid receptor (KOR) and GAPDH for nuclear/debris (N), membranous (Mb) and cytosolic (Cyto) fractions, respectively. T: total extract. (**b**) Quantification of GR in each fraction of LβT2 cells treated or not (V) with Dex, in comparison with T. Results are expressed as means ± SEM fold stimulation when compared to T, one-way ANOVA with Newman-Keuls Multiple Comparison Test, *n* = 4 experiments, **p < 0.01, and ***p < 0.001. (**c**) Upper panel, LβT2 cells were pre-incubated with 2-Br prior to cell fractionation. Equal amounts of proteins were analyzed by WB. *n* = 3 experiments. 2-Br: 2-bromopalmitate. Lower panel, LβT2 cell lysates were subjected to ABE assay after GR immunoprecipitation, followed by SDS-PAGE with the minus (−) and plus (+) HAM samples. Palmitoylation was detected by WB comparing streptavidin (Strept) signal to GR one. *n* = 3 experiments. (**d**) HEK293 cells were transfected with GR_Wt_, GR_C/A_ or stathmin 1-GFP (St1). Cell lysates were subjected to the ABE protocol followed by SDS-PAGE as described in (**c**). Strept signals detecting palmitoylation were normalized to the level of immunoprecipitated Flag-GR or GFP-St1 detected on the same membrane. Graph shows the average normalized densitometry data (Strept signal divided by anti-Flag or anti-GFP signals) displayed as fold induction of (+) HAM/(−) HAM control samples. One-way ANOVA with Dunnett’s posttest (*n* = 4 experiments, *p < 0.05, **p < 0.01). (**e**) LβT2 cells were pretreated with 2-Br or Nifedipine (Nif) respectively, and then treated with either Cort or Cort-BSA for 5 min. Cell lysates were subjected to WB with anti-CaMKII, anti-p-CaMKII, and anti-α-tubulin antibodies. Results are expressed as means ± SEM fold stimulation when compared to vehicle (V). One-way ANOVA with Dunnett’s post-test, *n* = 3 experiments, ***p < 0.001 when compared to V. Cropped blots are shown. Full length WBs are presented in Supplementary Fig. S4d,e.

This observed rapid membrane-initiated signaling strongly suggests the existence of a membrane form of GR (MbGR) in LβT2 gonadotrope cells. Therefore, we investigated the subcellular localization of endogenous GR in LβT2 cells by cell fractionation, each fraction being analyzed by Western blotting. As illustrated in Fig. 3a using classical GR antibodies (M20), a single band at approximately 100 kDa corresponding to GR was detected in total LβT2 cell lysates in the absence (Vehicle, V) or presence of Dex, as well as in all three subcellular compartments (Nuclei/Debris (N), Cytoplasmic (Cyto) and Membranous (Mb) fractions). Quantification of GR signal in each subcellular compartment in the vehicle condition further confirmed that GR was mainly located in the cytoplasm (ratio Cyto/Total = 2 ± 0.32). Noteworthy, our results demonstrate that a significant amount of GR was also recovered in the membranous fraction of LβT2 cells (ratio Mb/Total = 0.9 ± 0.2) (Fig. 3b). A membrane form of GR is therefore present in gonadotrope cells. Such a form could also be detected in various GC-sensitive cell lines originating from various tissues (Supplementary Fig. S3), clearly demonstrating that although the bulk of GR is in the cytoplasm and nucleus, a significant proportion of GR is tethered or anchored to cell membranes of GC-sensitive cells. To test whether subcellular GR localization could be modified in the presence of ligand, LβT2 cells were incubated with 10^−7^ M Dex for 30 min before cell fractionation. As expected, nuclear GR was significantly increased upon Dex treatment, while cytoplasmic signal was concomitantly reduced (Fig. 3b), in agreement with the hormone-dependent GR translocation from cytoplasm to the nucleus. Importantly, the presence of ligand did not modify the intensity of MbGR signal suggesting that acute Dex exposure does not affect MbGR level (Fig. 3b).

Since palmitoylation of ER was previously shown to be the major determinant for ER residence at the plasma membrane^34^, we evaluated the effect of the palmitoylation inhibitor 2-bromopalmitate (2-Br) on MbGR localization. As illustrated in Fig. 3c (upper panel), overnight incubation of LβT2 cells with 100 μM 2-Br totally abolished MbGR localization without modifying the presence of the membrane marker, the κ-opioid receptor (KOR), in the membranous fraction (ratio Mb/Total = 1.32 ± 0.2 vs 1.43 ± 0.3), strongly suggesting that endogenous GR is palmitoylated. To further confirm GR palmitoylation, we performed an ABE assay^35^ on LβT2 cells. During the ABE assay, unmodified free cysteines of immunoprecipitated GR were alkylated with NEM. Next, hydroxylamide (HAM) specifically cleaved palmitates from cysteines and then exposed these sites for biotinylation. Once the sites were labeled, palmitoylation was analyzed by SDS-PAGE and Western blotting using fluorophore-conjugated streptavidin (Strept); Strept signals correspond to protein biotinylation of palmitoylation sites. The same membrane was also used for Western blotting with an anti-GR antibody (Fig. 3c, lower panel). As shown in Fig. 3c (lower panel), Strept signals were only revealed after HAM treatment condition (+), despite an equivalent immunoprecipitation efficiency between the two conditions. This result confirms that GR is clearly palmitoylated in LβT2 cells and demonstrates that GR is addressed to the plasma membrane through palmitoylation. Next, we assessed whether the palmitoylation site of GR was conserved with the one described for ER, AR and PR^28^. We mutated the ER corresponding cysteine residue of GR (Cys671) into Alanine (C/A) on a Flag-form of GR and performed an ABE assay after overexpressing GR_Wt_ or GR_C/A_ in HEK293 cells. A GFP-form of stathmin 1 (St1) known as a soluble non-palmitoylable protein^36^ was used as a negative control. Strept signal was normalized to the amount of tagged protein detected on the same blot (Fig. 3d). As shown in Fig. 3d, GR_Wt_ treated with HAM was significantly biotinylated and specifically labeled by Strept (about 2.7-fold induction compared to the absence of HAM), indicating that overexpressed GR is also palmitoylated in HEK293 cells. Importantly, mutation of Cys671 into Ala (GR_C/A_) led to a GR mutant that remained palmitoylated at an equivalent level as the GR_Wt_ (Strept/Flag signals between “−HAM” and “+HAM” increased by 3.6 ± 1.5 -fold *vs* 2.7 ± 1.2 -fold, respectively). Immunoprecipitated stathmin 1 (St1) was not labeled by Strept providing additional support for GR palmitoylation. Thus, in sharp contrast with ER, the putative palmitoylation consensus site Cys671 is not involved in the GR membrane targeting.

We also tested whether palmitoylated GR was responsible for rapid GC-induced signaling. As shown in Fig. 3e, overnight pretreatment of LβT2 cells with 100 µM 2-Br totally abolished both Cort- and Cort-BSA-induced CaMKII phosphorylation when compared to vehicle. Total CaMKII levels were not modified by 2-Br treatment (not shown). These data unambiguously demonstrate that the palmitoylated form of GR triggers GC rapid signaling on CaMKII phosphorylation.

Calmodulin-associated calcium promotes CaMKII autophosphorylation and subsequently CaMKII activation^37^. Therefore, we examined the mechanism by which GC would increase intracellular calcium concentration by following the effects of an L-type calcium channel blocker (Nifedipine) on GC induced CaMKII phosphorylation. As show in Fig. 3e, 1h pretreatment with 10 μM Nifedipine (Nif) prevented CaMKII phosphorylation measured after 5 min of Cort or Cort-BSA treatment. Nif did not modify total levels of CaMKII (not shown). Overall, GC rapidly promote membrane-initiated signaling involving a palmitoylated GR that elicits calcium entry through L-type calcium channel that participates to CaMKII phosphorylation and leads to synapsin phosphorylation.

### GC affect GnRH signaling to reduce LH secretion

**Figure 4:**
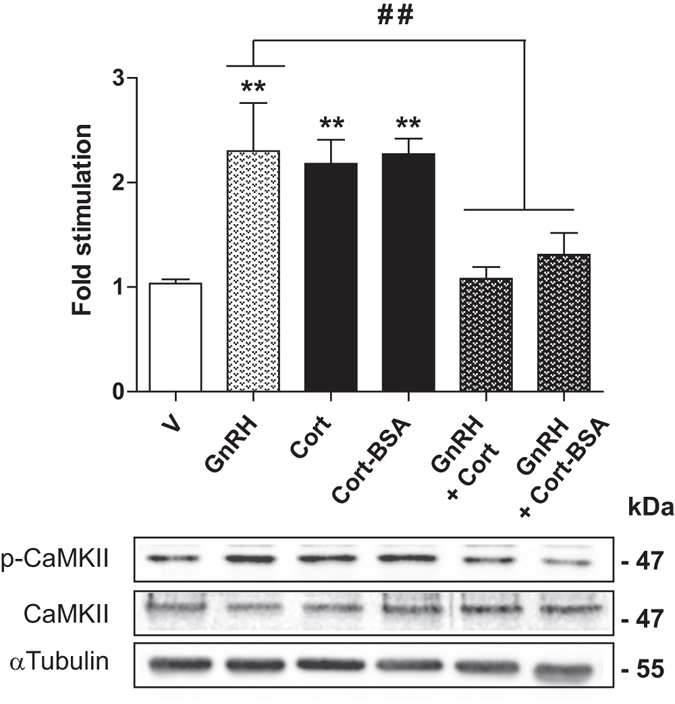
GC prevent GnRH-induced CaMKII phosphorylation. LβT2 cells were treated or not (V) with GnRH (10^−8^ M), Cort (10^−7^ M), Cort-BSA (10^−7^ M) or co-treated with either GnRH and Cort or GnRH and Cort-BSA for 5 min. Cell lysates were subjected to WB with anti-CaMKII, anti-p-CaMKII, and anti-α-tubulin antibodies. Cropped blots (full length WBs are presented in Supplementary Fig. S4e) are shown in the figure and are representative of 3 independent experiments. Results are expressed as means ± SEM fold stimulation when compared to vehicle. One-way ANOVA with Dunnett’s post-test, **p < 0.01. ^##^ *p* < 0.01 comparing GnRH with both co-treatments.

GnRH has been described to rapidly stimulate CaMKII phosphorylation (by about 2.5-fold) in rodent pituitary cells^37^, thus regulating gonadotropin subunit transcription. Therefore, we investigated whether GC could influence GnRH-induced CaMKII phosphorylation in LβT2 cells. Figure 4 shows that treatment with GnRH (10^−9^ M) for 5 min increased by about 2.3-fold p-CaMKII levels compared with vehicle. These phosphorylation levels were in the same range than those measured after Cort or Cort-BSA exposure alone (Fig. 4). Interestingly, co-treatment with GnRH and Cort or Cort-BSA for 5 min prevented GnRH-induced CaMKII phosphorylation. Total CaMKII levels remained unchanged after any of these hormonal stimulations. These data strongly indicate that GC affect GnRH signaling by preventing GnRH-dependent CaMKII phosphorylation in LβT2 cells.

**Figure 5:**
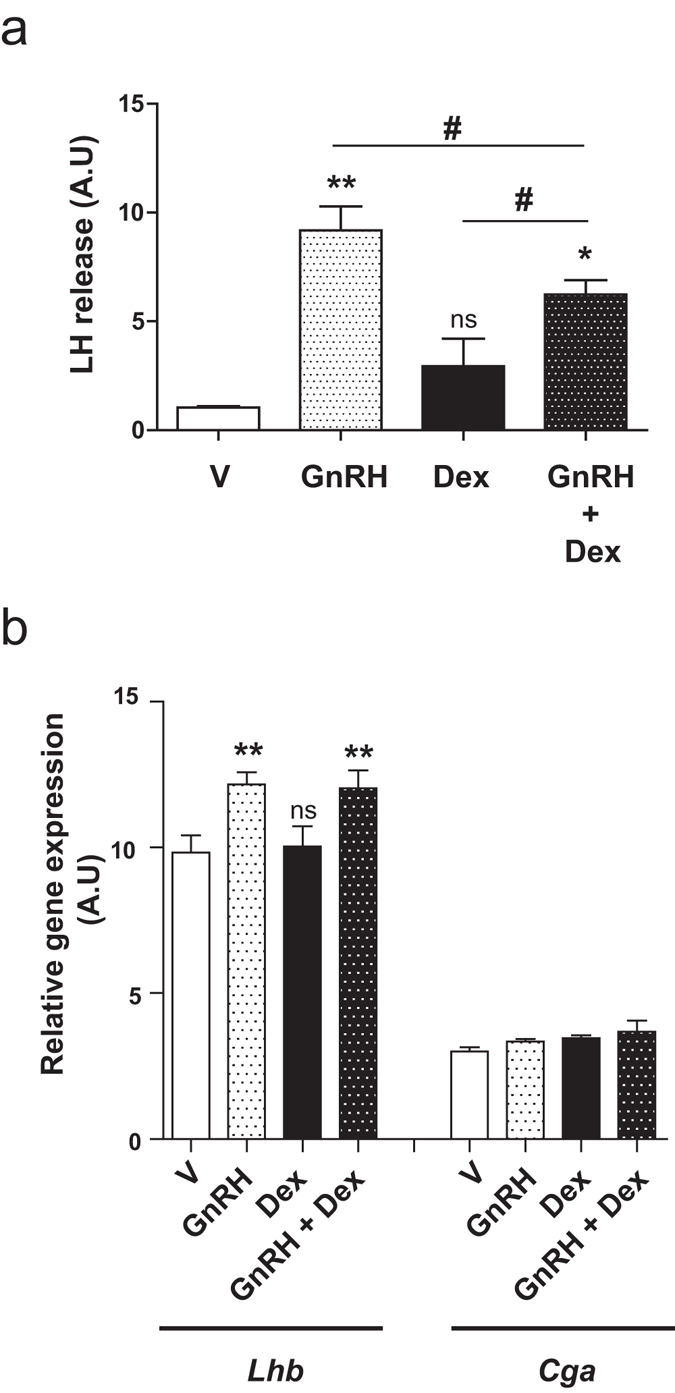
GC prevent GnRH-induced LH secretion in mice pituitary explants. (**a**) Pituitary explants were either treated with GnRH (10^−8^ M), Dex (10^−7^ M) or co-incubated with these two hormones for 1 h at 37 °C. LH concentrations were assayed in the culture supernatants as described in Methods. LH release after hormonal treatment was quantified (ng/ml) in comparison with LH amount measured in basal condition (pre-stimulation). GnRH significantly induced LH release when compared to vehicle. This effect significantly decreased after co-incubation of GnRH with Dex. One-way ANOVA with Dunnett’s post-test, *p < 0.05, **p < 0.01. ^#^ *p* < 0.05 comparing GnRH and Dex co-treatment with each individual treatment. (**b**) LβT2 were treated with GnRH (10^−8^ M), Dex (10^−7^ M) or co-incubated with these two hormones for 1 h at 37 °C before RT-qPCR to follow the expression of genes encoding the common α-glycoprotein subunit (*Cga*) and the β-subunit of LH (*Lhb*). Data show relative expression (arbitrary units) normalized with the reference gene *36b4*. Within 1 h, GnRH significantly stimulated *Lhb* expression (by approximately 1.24-fold above basal), whereas Dex did not have any effects either alone or associated with GnRH. One-way ANOVA with Newman-Keuls Multiple Comparison posttest, n = 3, **p < 0.01.

Since CaMKII was also described to be involved in insulin secretion^38^, we wondered whether this kinase could also modulate LH secretion. Because detection of LH release in LβT2 culture media was not possible at early time points due to the low secretory activity of this cell line, we tested the effect of GC treatment on LH release from pituitary explants. The integrity of the pituitary explants was controlled at the end of the procedure by histological approaches (not shown). Mouse pituitary explants were pre-incubated for 30 min in DMEM at 37 °C before stimulation to measure basal level of LH content present in culture supernatants of each explant. Culture media was then replaced by fresh media containing either GnRH (10^−8^ M), Dex (10^−7^ M) or both hormones for 1 h. LH contents were subsequently assayed from culture supernatants. As anticipated, Fig. 5a shows that GnRH strongly increased (by about 9-fold) LH secretion. In contrast, Dex did not induce any significant LH release. Noteworthy, co-incubation of pituitary explants with both GnRH and Dex significantly reduced LH release by 1.5-fold when compared to GnRH alone (Fig. 5a).

Since Dex binds both membrane and non-membrane GR, one could expect Dex to exert genomic activities that could contribute to Dex inhibitory effect on GnRH-induced LH release. Therefore, to investigate a possible impact of Dex on the expression of genes encoding the common α-glycoprotein subunit (*Cga*) and the β-subunit of LH (*Lhb*), we performed RT-qPCR on total RNA extracted from LβT2 cells treated with GnRH (10^−8^ M), Dex (10^−7^ M) or both hormones for 1 h (Fig. 5b). GnRH treatment significantly increased *Lhb* expression (by about 1.24-fold) whereas Dex alone or associated with GnRH was ineffective on *Lhb* or *Cga* expression at this very early time point when compared to vehicle or GnRH respectively (Fig. 5b). In contrast and as shown in Supplementary Fig. S1, under the same experimental conditions, Dex was able to regulate *Sgk1* expression; after 1 h treatment, Dex induced a 1.7-fold induction of *Sgk-1* mRNA levels. These results confirm that Dex blockade of GnRH-induced LH secretion involves rapid non-genomic mechanisms.

Altogether, these results demonstrate that GC rapidly regulate GnRH-induced CaMKII phosphorylation as well as impact on GnRH-dependent LH secretion.

## Discussion

Stress-induced disturbance of reproductive function is well described in many species including humans^3, 5, 39^, and is typically associated with an activation of the hypothalamic-pituitary adrenal axis, resulting in an increased secretion of GC. GC act directly *via* GR in the pituitary gland^40^ to elicit a rapid decrease (≤30 min) in responsiveness to GnRH, independent of changes in GnRH-R expression^7^, leading to a strong reduction of gonadotropin release^41^. Non-genomic mechanisms activated by a membrane anchored GR (MbGR) have been proposed to explain such rapid GC events.

In the present study, we provide evidence that GC rapidly trigger CaMKII and Syn phosphorylation in gonadotrope LβT2 cells. We demonstrated that Cort elicited a rapid (significant up to 15 min) and transient effect on CaMKII phosphorylation whereas a significant induction of p-Syn was detected up to 60 min after GC exposure. *Ex vivo*, by using pituitary explants, we demonstrated that Dex also induces phosphorylation of CaMKII and Syn after 30 min treatment, likewise what observed *in vitro*, in gonadotrope LβT2 cells exposed to Cort. Interestingly, LβT2 cells treated with a membrane impermeable GC (Cort-BSA) induced similar phosphorylation patterns of p-CaMKII and p-Syn than those obtained with Cort. However, p-CaMKII rapidly returned to basal levels indicating dephophorylation processes after Cort treatment, as opposed to Cort-BSA. These data suggest a negative regulatory feedback thereby GC, acting genomically through unknown genomic events, might desensitize its activated-signaling pathway more rapidly and efficiently than Cort-BSA. In addition and contrary to Dex or Cort, no induction of *Sgk-1* expression was observed after 1 h or 4 h of treatment with Cort-BSA, providing additional support that Cort-BSA effects are clearly only mediated by membrane-initiated non-genomic processes in LβT2 cells. Our results clearly differ from those reported in another study that showed genomic activities with Cort-BSA at a much higher concentrations (3 μg/ml *vs* 6.6 ng/ml in the present study) on human lymphoma cells^42^. In addition, contrary to other cell types^14, 16, 17, 43^, Dex was unable to induce phosphorylation of ERK1/2 or Src indicating that, depending on the cellular context and the nature of the associated signalosome, a wide variety of signal transduction pathways may be initiated. From plasma membrane of LβT2 cells, GC may promote L-type Calcium channel activation that triggers the calcium increase necessary for CaMKII phosphorylation and the resulting downstream signaling cascade.

To further understand this rapid signaling in gonadotrope LβT2 cells, we identified a MbGR and provided evidence that palmitoylation, a reversible posttranslational modification, promotes GR membrane targeting. We also demonstrated a direct involvement of palmitoylated form of GR in triggering CaMKII phosphorylation and activation, after Cort or Cort-BSA treatment in LβT2 cells. Contrary to others who used an hypothalamic cell line 4B^33^ and performed metabolic labeling presenting low sensitivity detection, we demonstrated that endogenous as well as overexpressed GR are truly palmitoylated. These data further support previous data performed in lymphocytes which showed that GR membrane targeting was prevented by a Golgi-disrupting agent (brefeldin A) treatment^19^, indicating that GR membrane attachment might take place in the Golgi where most of protein palmitoylation occurs^44^. Interestingly yet surprisingly, we demonstrated by site directed mutagenesis that ER and GR do not share the equivalent palmitoylation site, arguing against a consensus palmitoylation site among steroid receptors. Further studies are required to identify GR palmitoylation sites. Contrary to ER whose membrane transport relies on caveolin-1, two studies demonstrated that caveolin-1 is not the limiting factor for membrane transport of GR, without ruling out the possibility that it is a component of the transport machinery^22, 42^. A recent study using several protein structure algorithms predicted that some steroid receptor ligand binding domains contain one or more transmembrane helices and/or pore lining regions that may be essential for their translocation to the plasma membrane^45^. Herein, we also show as already described by others^23^ that Dex does not modify the level of MbGR in gonadotrope LβT2 cells. This finding contrasts though with the observation that Cort promoted MbGR level increase in hypothalamic cell line 4B^16^, and suggests that the regulation of MbGR might be dependent on the cell type.

In pituitary, GnRH-R is primary expressed in gonadotrope cells. Upon GnRH binding to GnRH-R, a wide range of intracellular signaling pathways including CaMKII phosphorylation is activated that ultimately regulates the synthesis and release of gonadotropins^46^. In the present study, we show that Cort or Cort-BSA rapidly induced phosphorylation of CaMKII and synapsin-I. However, in the presence of GnRH, GC rapidly prevented GnRH-induced CaMKII phosphorylation, by preventing calcium mobilization probably in localized sub-cellular regions, and efficiently reduced LH release by gonadotrope cells. Contrary to GnRH, Dex alone or associated with GnRH does not rapidly affect *Lhb* or *Cga* expression demonstrating that Dex inhibition of GnRH-induced LH secretion involves rapid non-genomic regulatory mechanisms.

These data suggest that during anestrus or in between GnRH pulses that could be widely spaced depending on the species, GC exert rapid non genomic signaling involving both CaMKII and synapsin-I, whereas during GnRH stimulation GC prevent GnRH-induced CaMKII phosphorylation that may account for the inhibitory effects of GC on LH secretion. Such paradoxical actions of GCs have already been described to depend on the timing, duration, and magnitude of GC exposure^47^. In the present situation, one could speculate that MbGR might interact with GnRH-R to regulate its associated signaling upon GnRH binding. GR and GnRH-R have been reported to co-localize with the lipid raft protein flotillin-1 at the plasma membrane, independently of the presence of ligands^23^. GnRH and GC were reported to synergistically inhibit cell proliferation *via* PKC activation and *Sgk-1* up-regulation in LβT2 cells^23^. Rapid non-genomic as well as genomic cross-talk mechanisms between the GnRH-R and GR signaling pathways have already been described in LβT2 cells^48^. GnRH may rapidly activate the unliganded GR *via* GnRH-R dependent phosphorylation involving MAPKs, leading to nuclear translocation and GR transactivation *via* a glucocorticoid response element. GC and GnRH may also modulate GnRH-R mRNA levels *via* a genomic mechanism involving GR binding to an AP-1 motif of the murine GnRH-R promoter^48^. In the present study, we propose another level of cross-talk mechanisms in LβT2 cells in which GC may regulate the level of GnRH-induced phosphorylation of CaMKII and synapsin-I that ultimately regulate LH release. We showed that Dex inhibited GnRH-induced LH release by pituitary explants. It remains to determine whether this inhibition is triggered by MbGR. GC have already been described to rapidly inhibit ACTH release in pituitary AtT-20 cells^49, 50^ or Cl^−^ secretion in human bronchial epithelial cells^18^. CaMKII is a multifunctional kinase that has been depicted to mediate insulin secretion^51^ by phosphorylating synapsin-I^38^, already known to control neurotransmitters release^52^. Synapsin-I has been described as an important molecular effector of the GR signaling pathway induced in hippocampus involved in increased stress-related memory^53^. We demonstrated in LβT2 cells that GC signaling pathways encompass both CaMKII and synapsin-I. However, in the presence of GnRH, GC do not trigger this signaling pathways any longer to ultimately prevent LH release. It remains to precisely decipher the molecular mechanisms by which GC and GnRH signaling cross-talk, even though such a task might be very difficult to explore *in vivo* given that both GC and GnRH signaling share several downstream effectors. In addition, owing to the fact that GR is expressed in almost all pituitary cells, GC action is not restricted to gonadotrope cells but also affect other cell lineage populations within pituitary.

Finally, our work brings new findings in GC field, unraveling novel understanding on how GR integrates plasma membrane allowing GC membrane-initiated signaling through L-type calcium channel activation, CaMKII and synapsin-I phosphorylation in gonadotrope cells. A novel conceptual advance in the cross-talk between GnRH and GC signaling pathways has been discovered that could ultimately regulate pituitary LH release. Future challenge should clarify the existence of other pathways between GnRH and GC signaling that could account for desensitization of gonadotrope cells to GnRH during stress.

## Methods

The antibodies and products used in the present study were purchased from the following sources:

Monoclonal anti–α tubulin (Sigma-Aldrich, St. Louis, MO, USA), Polyclonal anti-p-CaMKII (T286) (sc-12886-R, Santa Cruz Biotechnology, CA, USA), Polyclonal anti-CaMKII (H300) (sc-13082, Santa Cruz Biotechnology), Polyclonal anti-p-synapsin Ia/b (S603) (sc-135708, Santa Cruz Biotechnology), Polyclonal anti-synapsin Ia/b (H170) (sc-20780, Santa Cruz Biotechnology), Polyclonal anti-p-Src and anti-Src (Cell Signaling Technology, Danvers, MA, USA), Monoclonal anti-Flag M2 (Sigma-Aldrich), Polyclonal anti-GR M20 (Santa Cruz Biotechnology), Polyclonal anti-Lamin A/C (Cell Signaling Technology), Monoclonal anti-GAPDH (Sigma-Aldrich), monoclonal anti-GFP (Roche Diagnostics, Mannheim, Germany), Polyclonal anti-κ-Opioid Receptor (Abcam, Cambridge, UK), RU486 (mifepristone) (Sigma-Aldrich), 2-bromohexadecanoic acid (Sigma-Aldrich), Dexamethasone (Dex) (Sigma-Aldrich), Corticosterone (Sigma-Aldrich), Corticosterone-BSA conjugate (Cusabio Biotech Co., CliniSciences, Nanterre, France), KN93 (Sigma-Aldrich), DMEM-glutamax (Dulbecco’s Modified Minimum Essential Medium)-glucose 4.5 g/l (Life Technologies, Saint-Aubin, France), Fetal calf serum (FCS) (Biowest, Nuaillé, France), Lipofectamine 2000 (Life Technologies), OptiMEM (Life Technologies), Hydroxylamine (Sigma-Aldrich), EZ-link biotin-BMCC ((1-biotinamido-4-[4′-maleimidomethyl) cyclohexanecarboxamido] butane) (Thermo Fisher Scientific, Rockford, IL, USA), *N*-ethylmaleimide (NEM) (Merck Millipore, Darmstadt, Germany) and *N*-methylmaleimide (NMM) (Sigma-Aldrich).

### Pituitary Culture

Adult male mice SV129 were decapitated and the whole pituitaries were rapidly collected and incubated in DMEM-glutamax medium at 37 °C in 5% CO_2_ for 2 h (equilibration) before hormonal treatment. This procedure was carried out in accordance with relevant guidelines and regulations following a protocol approved by the Animal Care Committee, Ministère de l’Agriculture, France (N°C94-043-12). All procedures were approved by the local ethics committee Consortium des Animaleries Paris Sud (CAPSud) (N°2012-021).

### Cell line culture and transfection

Cell lines, LβT2^54^, GT1-7^55^ and HEK293 cells (obtained from American Type Culture Collection (ATCC; Manassas, VA, USA)) were cultured in DMEM containing 10% fetal calf serum (FCS) supplemented with 100 U/ml penicillin (Life technologies), 100 μg/ml streptomycin (Life technologies), at 37 °C in 5% CO_2_. KC3AC1 cells^56^ were seeded on collagen I-coated dishes and routinely cultured at 37 °C in 5% CO_2_ as previously described^56^. mhATF3F cells^57^ were cultured in DMEM/F12 (1:1) medium supplemented with 100 U/ml penicillin, 100 μg/ml streptomycin, 10 nM insulin, 2 mM glutamine, 1 µM Dex, 30 nM sodium selenite, 1 µM 3,4,5-tri-iodo D-thyronine and 5% FCS. All cells were serum-starved for 24 h prior to drug treatment. HEK293 cells were transfected with Flag-GR using Lipofectamine 2000 according to the manufacturer’s instructions (Life Technologies). Twenty four hours after transfection, cells were used for ABE assays.

### Construction of Flag-GR Wt or C/A mutant

Mouse GR cDNA was amplified by PCR using pSV2-GR plasmid, a generous gift of E. D. Sanchez (Dept Pharmacology, University of Toledo College of Medicine, Toledo OH 43614, US)^58^, and specific primers (Forward: 5′-GATCTATGGACTCCAAAGAATCC-3′, Reverse: 5′-CTAGATCATTTCTGATGAAACAGAAGC-3′) containing *BgIII* and *Xbal* restriction sites to facilitate cloning into the pFLAG-CMV2 vector (Sigma-Aldrich) leading to a N-terminal Flag form of GR. For Flag-GR C/A mutant, specific cysteine residue was mutated into alanine (Forward primer: 5′-GAAGAGTATCTCGCTATGAAAACCTTAC-3′; Reverse primer: 5′-CTGATTATTAATGAGCAGAGA-3′, mutated codons are underlined) by Quick change Site-Directed Mutagenesis Kit (Agilent Technologies). All plasmid sequences were verified by DNA sequencing.

### Cell fractionation

Subcellular compartments of each cell line were separated according to specific protocols as previously described^59^. Cells were homogenized in a Dounce B homogenizer in buffer composed of 10 mM Hepes, 250 mM sucrose, 1 mM EDTA, pH 7.4, with an anti-protease cocktail (Sigma-Aldrich) at 4 °C and fractionated by differential centrifugation. The initial homogenate was first centrifuged at 850 × *g* for 5 minutes at 4 °C to obtain a pellet corresponding to nuclear (N) fraction that could also contain cell debris. Then, the post-nuclear supernatant was centrifuged at 10 000 × *g* for 10 minutes at 4 °C to remove mitochondria. The resulting supernatant was finally centrifuged at 400 000 × *g* for 6 minutes at 4 °C to obtain the soluble (supernatant, Cyto) and membrane (pellet, Mb) fractions. Each pellet was washed 3 times before resuspension in RIPA (50 mM Tris-HCl pH 7.4, 150 mM NaCl, 0.5% sodium deoxycholate, 0.1% SDS, 1% Triton, protease inhibitors cocktail (Sigma-Aldrich).

Equal amounts (50 μg) of each fraction were analyzed by SDS–PAGE. The purity of each fraction was assessed by immunoblotting for specific protein markers: lamin A/C at approximately 70 kDa in the nuclear fraction, a 37 kDa band for glyceraldehyde 3-phosphate dehydrogenase (GAPDH, cytosol), and a band 45 kDa κ-opioid receptor (KOR, membranes). These analyses showed little contamination between compartment fractions indicating that the purity of the different fractions. Quantification of the membrane and the soluble pools of GR was analyzed and compared to the total extract (T) and expressed as a percentage of the total.

### Protein extraction

After drug treatment, cells were scraped in RIPA (50 mM Tris-HCl pH 7.4, 150 mM NaCl, 0.5% sodium deoxycholate, 0.1% SDS, 1% Triton, 10% Glycerol, protease inhibitors cocktail (Sigma-Aldrich) and phosphatase inhibitors (Sigma-Aldrich). Pituitaries were homogenized in RIPA with TissueLyser LT homogenizer (Qiagen, Les Ulis, France). After centrifugation (14,000 × g, 4 °C for 15 min) protein content was determined by bicinchoninic acid assay (Thermo Fisher Scientific).

### Western Blot analysis

Equal protein amounts (50 μg) were resolved by either 7.5% or 10% SDS-PAGE and then transferred to nitrocellulose membrane (Whatman Schleicher & Schuell, Dassel, Germany). Blots were incubated for 1 h at room temperature (RT) in a blocking buffer (5% fat-free milk or 5% BSA in 0.2% Tween 20 phosphate-buffered saline (PBS-T) or in 0.2% Tween 20 Tris-buffered saline (TBS-T) depending on the nature of the first antibody, before an overnight incubation at 4 °C with primary monoclonal or polyclonal antibodies as indicated. After extensive washes with PBS-T or TBS-T, blots were incubated with an IRDye 800-conjugated affinity purified anti-rabbit IgG second antibody (Perbio Science, Brebières, France) and an IRDye 680- conjugated affinity purified anti-mouse IgG second antibody (Perbio Science) for 1 h at RT. After washes, proteins were visualized with an Odyssey-Fc apparatus (Li-Cor, Lincoln, NE, USA). Specific signals for different proteins were normalized by the infrared fluorescence of α-tubulin followed by total CaMKII or total Syn or total ERK or total Src signals as determined by densitometry using the Image Studio software (Li-Cor). All Western blots shown are representative of what was observed in at least three independent experiments, excepted in Supplementary Fig. S2 (2 independent experiments).

### RT-qPCR

RT-qPCR analysis was undertaken using the Power SYBR Green PCR Master Mix (Life Technologies) with primers (*Sgk-1* Forward: 5′-TCACTTCTCATTCCAGACCGC-3′, Reverse: 5′-ATAGCCCAAGGCACTGGCTA-3′; *Lhb* Forward: 5′-ATCACCTTCACCACCAGCAT-3′, Reverse: 5′ GACCCCCACAGTCAGAGCTA-3′; *Cga* Forward: 5′ GCTGTCATTCTGGTCATGCT-3′, Reverse: 5′-GAAGCAACAGCCCATACACT-3′; *36b4* Forward: 5′AGCGCGTCCTGGCATTGTCTGT-3′, Reverse: 5′-GGGCAGCAGTGGTGGCAGCAGC-3′) and a StepOne Real-Time PCR System (Life Technologies). Expression levels of *Sgk-1*, *Lhb* and *Cga* analyzed by qPCR were normalized relative to levels of *36b4* mRNA, Cga coding for the alpha subunit of LH and FSH.

### ABE assay

LβT2 as well as HEK293 cells transfected with Flag-GR (GR) or Stathmin 1-GFP (St1) were lysed in presence of N-Ethylmaleimide (NEM). GR palmitoylation was assessed using the acyl biotin exchange (ABE) protocol as previously described^35^. The principle of this assay was to biotinylate palmitoylation sites after specific cleavage by hydroxylamide (HAM) on immunoprecipitated GR using either EZview Red anti-FLAG M2 agarose (Sigma-Aldrich) or anti-GR (M20) or GFP with anti-GFP associated with 50% slurry of protein G-coated sepharose beads (Roche Diagnostics). Once labeling sites was achieved, palmitoylation was analyzed by SDS-PAGE followed by Western blotting using Streptavidin-IRDye800CW conjugated (Rockland Immunochemicals, Gilbertsville, PA) as well as an anti-GR or anti-Flag or anti-GFP antibody. Streptavidin signals were normalized to the amount of Flag-GR protein present on the blot.

### LH assay

Whole pituitaries were cultured in DMEM for 2 h at 37 °C for equilibration. The medium (200 μl) was replaced by fresh medium for 30 min at 37 °C and collected for LH assay (pre-stimulation). Medium containing ethanol (vehicle), GnRH (10^−8^ M), Dex (10^−7^ M) or both hormones were used to treat pituitaries for 1 h at 37 °C. Culture supernatants were then collected to measure LH content. LH concentration in culture media was determined using an ELISA method as previously described^60^ with reagents supplied by Dr Parlow (NHPP, Harbor-UCLA, CA, USA). Briefly, micro-titration plates (High binding, Greiner Bio-one) were coated overnight at 4 C with 10 ng of purified rat LH-I-10 diluted in carbonate buffer. Rat LH-RP3, used as standard, and media were incubated with anti-rat LH-S11 (dilution 1:4,000 overnight at 4 °C). Plates were then rinsed with PBS containing 1% BSA and 0.1% Tween20 for 1 h at RT and washed with PBS-0.1% Tween20 before addition of standards and samples for competition binding (2 h at 4 °C). After removal of unbound material, phosphatase alkaline-labeled secondary antibody (dilution 1: 2,000, Thermo Scientific, France) was added for 1 h at 4 °C and phosphatase alkaline activity was revealed with SigmaFast pNPP reagent. The minimum detectable LH concentration was 0.2 ng/ml and the inter-assay coefficient of variation were <10%. LH release after hormonal treatment was quantified in comparison with LH amount measured in basal condition (pre-stimulation).

### Statistical Analysis

Means ± SEM of n experiments of measurements were analyzed for significant differences by one-way ANOVA using Prism 6 (GraphPad) software followed by appropriated posttests. Means are assumed to be significantly different if *p* < 0.05.

## Electronic supplementary Material

Supplementary information
