# Supplementary material for: A novel non genomic glucocorticoid signaling mediated by a membrane palmitoylated glucocorticoid receptor cross talks with GnRH in gonadotrope cells
Source: Sci Rep. 2017 May 8;7:1537. doi: 10.1038/s41598-017-01777-2 (PMC5431531; doi:10.1038/s41598-017-01777-2)
Supplement: Supplementary file 1 — Supplementary information [file 41598_2017_1777_MOESM1_ESM.pdf]

A novel non genomic glucocorticoid signaling mediated by a membrane palmitoylated glucocorticoid receptor cross talks with GnRH in gonadotrope cells

Mohsen Ayrout[1], Violaine Simon[2], Valérie Bernard[1], Nadine Binart[1], Joëlle Cohen-Tannoudji[2], Marc Lombès[1] and Stéphanie Chauvin[1,*]

Supplementary information

Supplementary Figure S1: Cort-BSA does not trigger genomic activities.

Total mRNAs were recovered from LβT2 cells treated for 1 to 4 h with Dex (10^{-7} M), Cort (10^{-7} M) or Cort-BSA (10^{-7} M). A well-known GC target gene, Serum and Glucocorticoid-regulated Kinase 1 (*Sgk1)* gene expression was evaluated by RTqPCR. Data show relative expression (arbitrary units) corrected for reference gene *36b4.* As anticipated, Dex as well as Cort significantly stimulated Sgk1 expression within 1 h by approximately 1.7 and 1.4-fold above basal, respectively. In sharp contrast, Cort-BSA was unable to induce *Sgk1* expression after 1 or 4 h of treatment, demonstrating that Cort-BSA was devoid of any uncoupled, free Cort and thus exclusively acted through membrane-mediated mechanism. Pretreatment with Cort-BSA for 20 min did not potentiate the transcriptional activity of Cort. One-way ANOVA with Tuckey's posttest, n=3, *$p < 0.05$ and **$p < 0.01$.

Supplementary Figure S2. GC do not stimulate either MAPK or Src signaling pathways in LβT2 cells.

(a) LβT2 cells were treated with GnRH (10^{-8} M) (g), Dex (10^{-7} M) (d) or co-treated with GnRH and Dex (gd) for 0 to 60 min. Cells lysates were subjected to western blotting with anti-ERK (in red) and p-ERK (in green). GnRH stimulated ERK phosphorylation within 5 min and maintained its effect after 60 min of treatment, whereas Dex alone or associated with GnRH did not have any effect on the level of ERK phosphorylation. Level of total ERK was unchanged during treatments. Results are representative of 2 separate experiments that gave similar results. (b) LβT2 cells were treated with Dex (10^{-7} M) for 0 to 60 min. Cells lysates were subjected to immunoblotting with anti-Src, p-Src and -α-tubulin antibodies. Dex did not significantly induce Src phosphorylation. Total Src level were unchanged during treatment. The immunoblot images are representative of 3 independent experiments. Results are expressed as means ± SEM fold stimulation when compared to vehicle. Statistical significance was assessed by One way ANOVA, Dunnett's post-test, n = 2; NS, no significant difference.

Supplementary Figure S3. GR is broadly detected in the membrane fraction of various murine cell lines.

LβT2 (gonadotrope cells), GT1-7 (hypothalamic cells), mhATF3F (hepatic cells) and KC3AC1 (kidney cells) were subjected to cell fractionation. Equal amounts (50 μg) of proteins from each fraction (Total (T), Nuclei/debris (N), Cytosolic (Cyto), Membranous (Mb)) were loaded on SDS-PAGE and subjected to immunoblotting with the anti-GR antibody. Results are representative of two separate experiments that gave similar results.

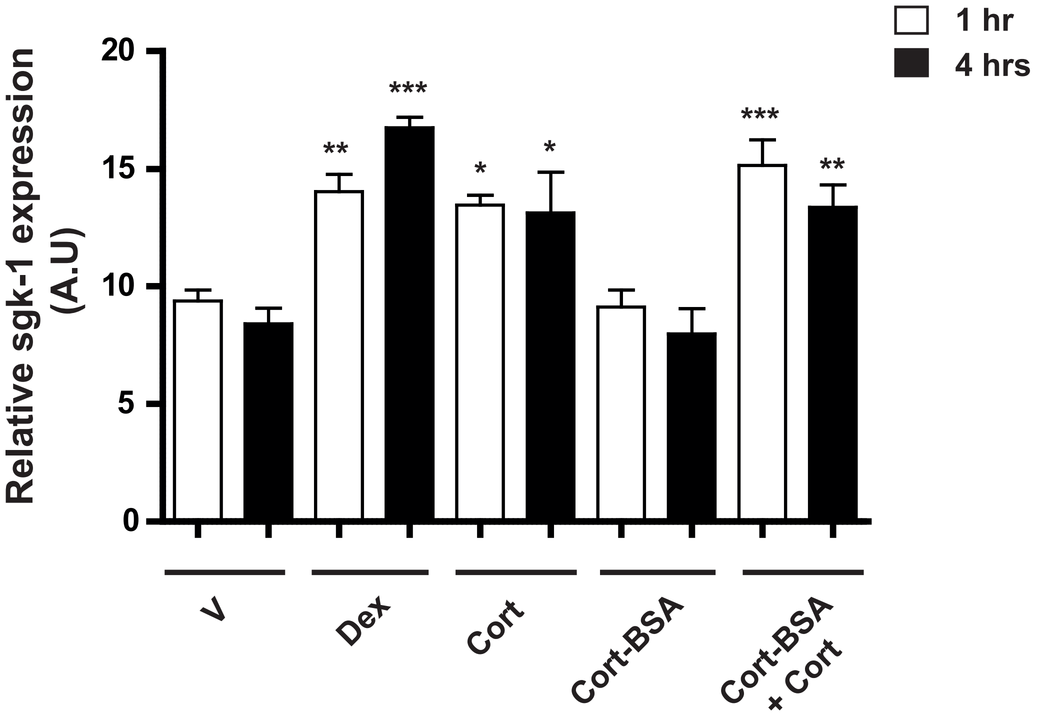

Supplementary Figure S1

a

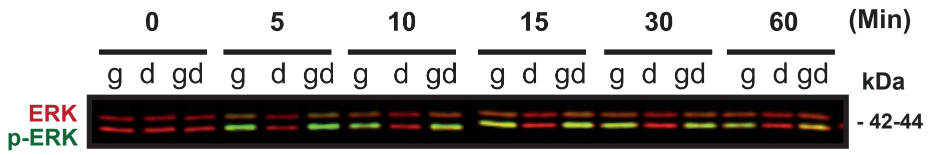

Treatments:
g: GnRH
d: Dex
gd: GnRH + Dex

b

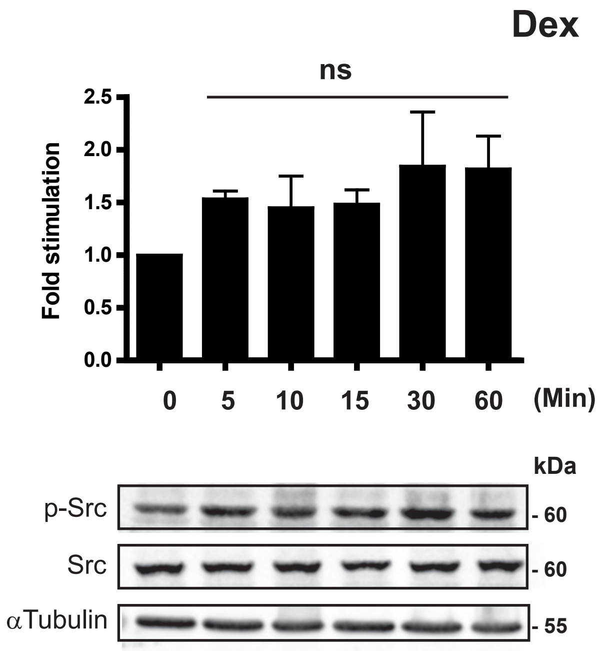

Supplementary Figure S2

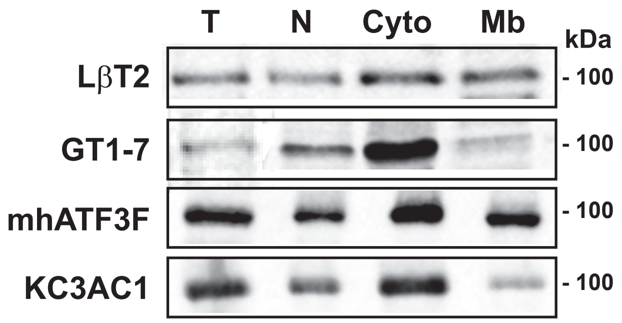

Supplementary Figure S3

Figure 1 a

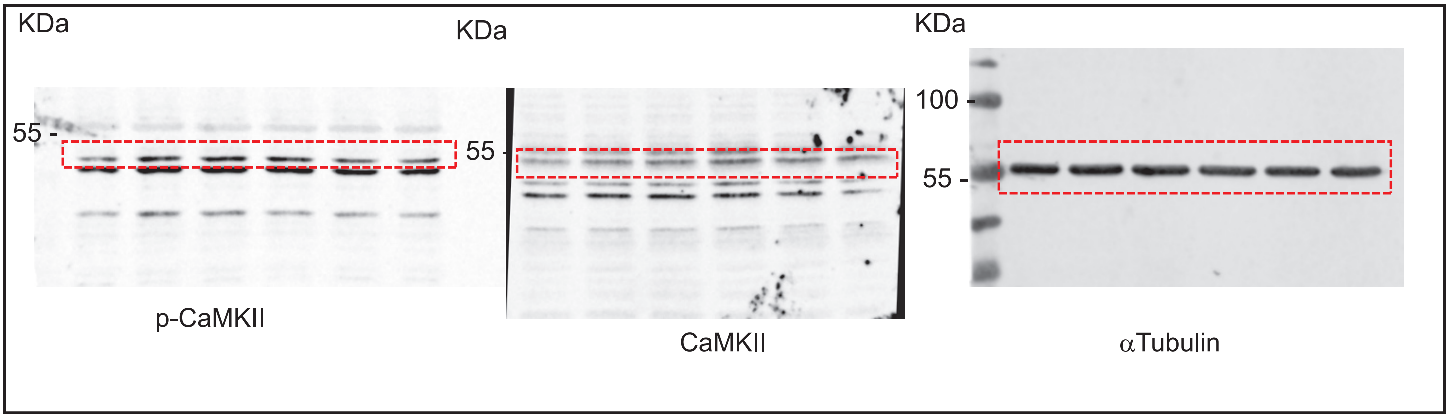

Figure 1 b

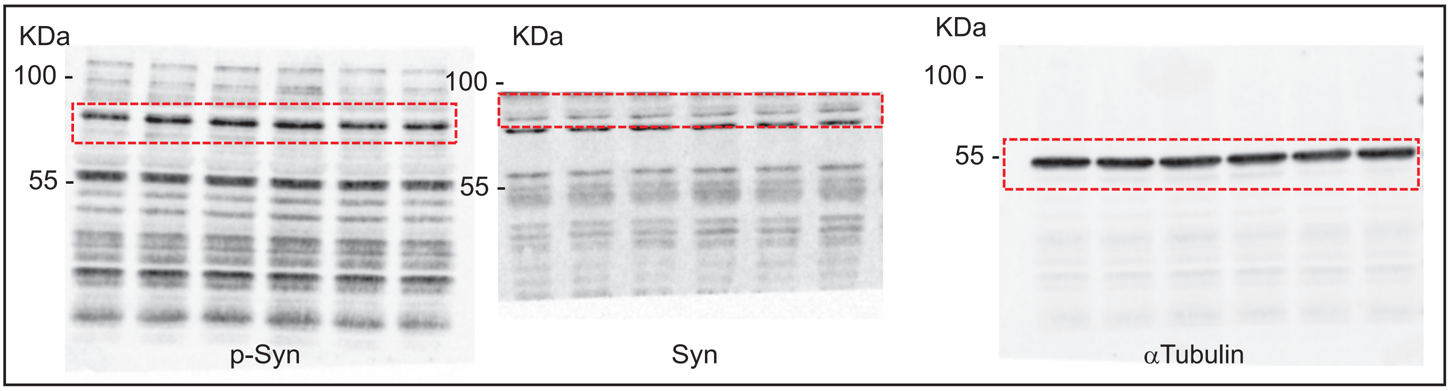

Figure 1c

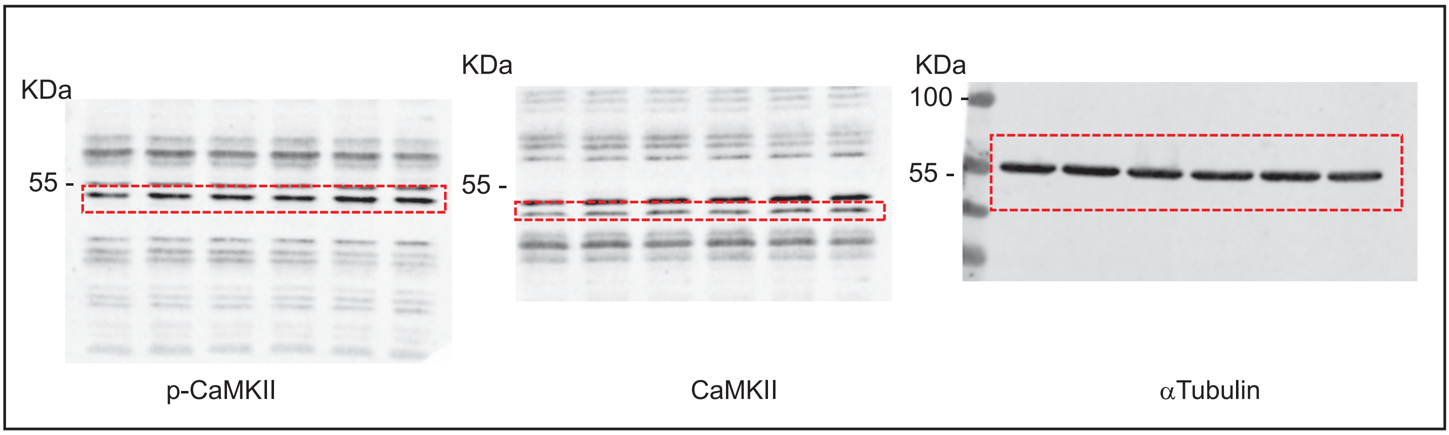

Figure 1d

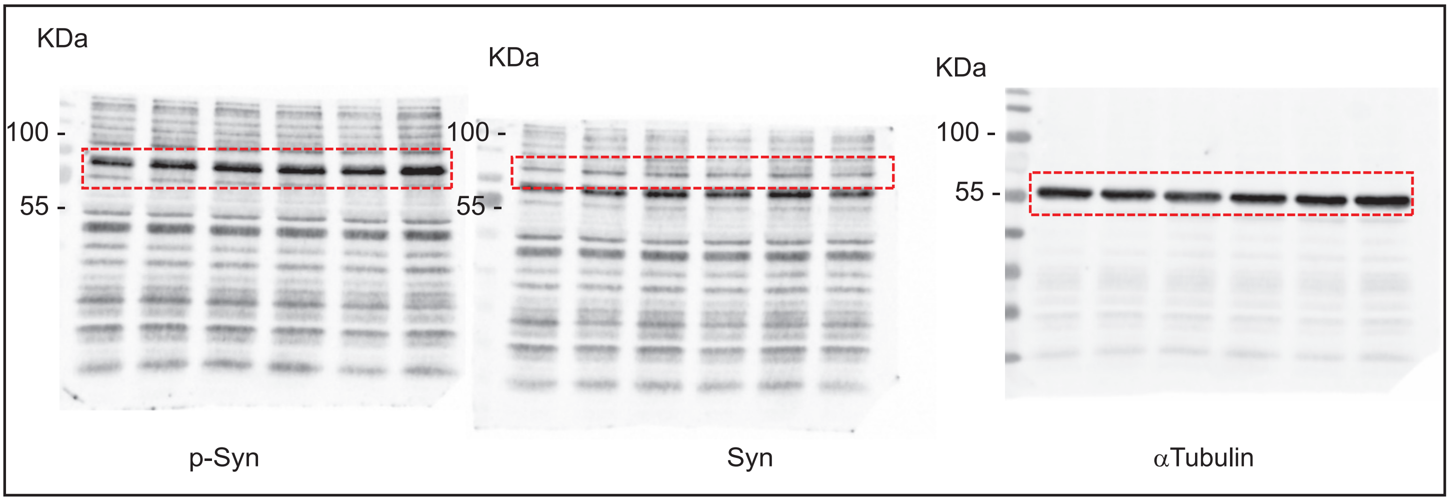

Supplementary Figure S4 a

Figure 1e

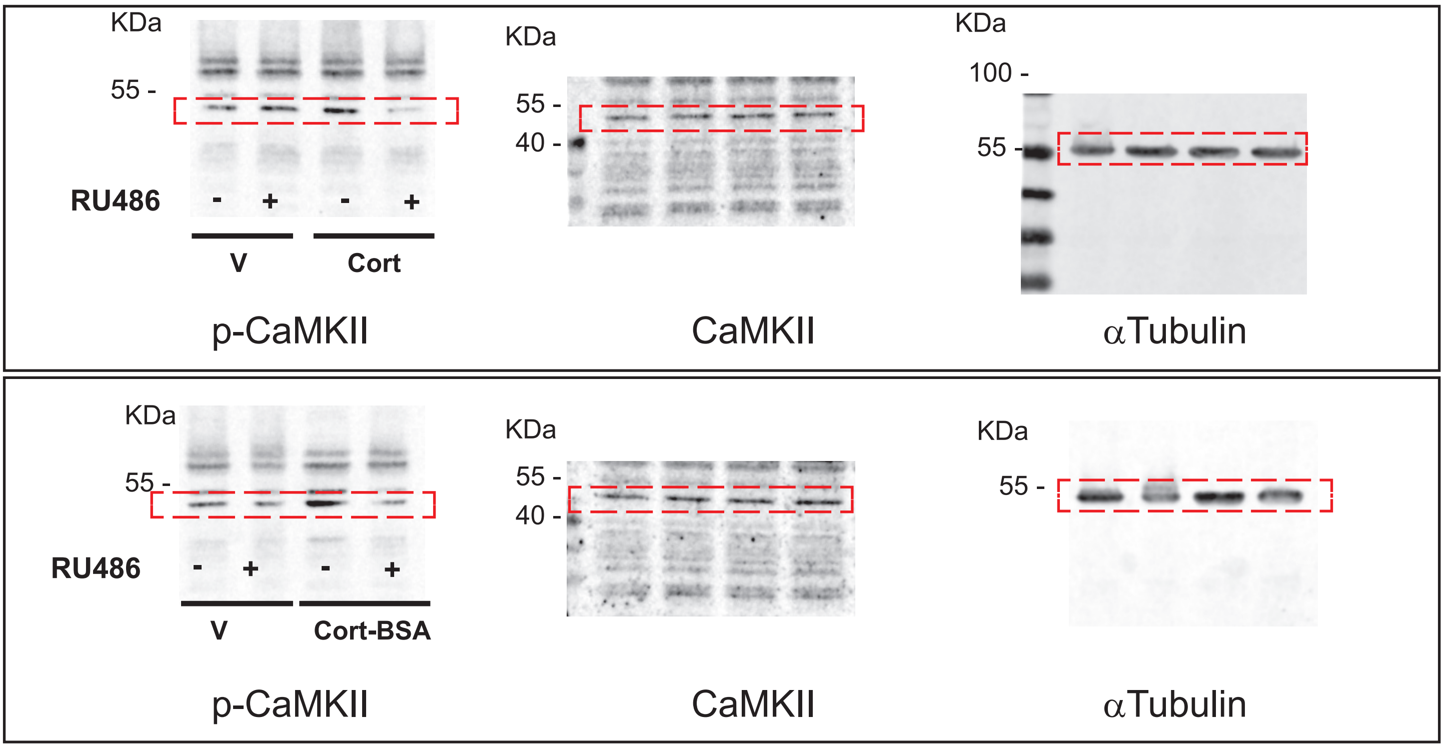

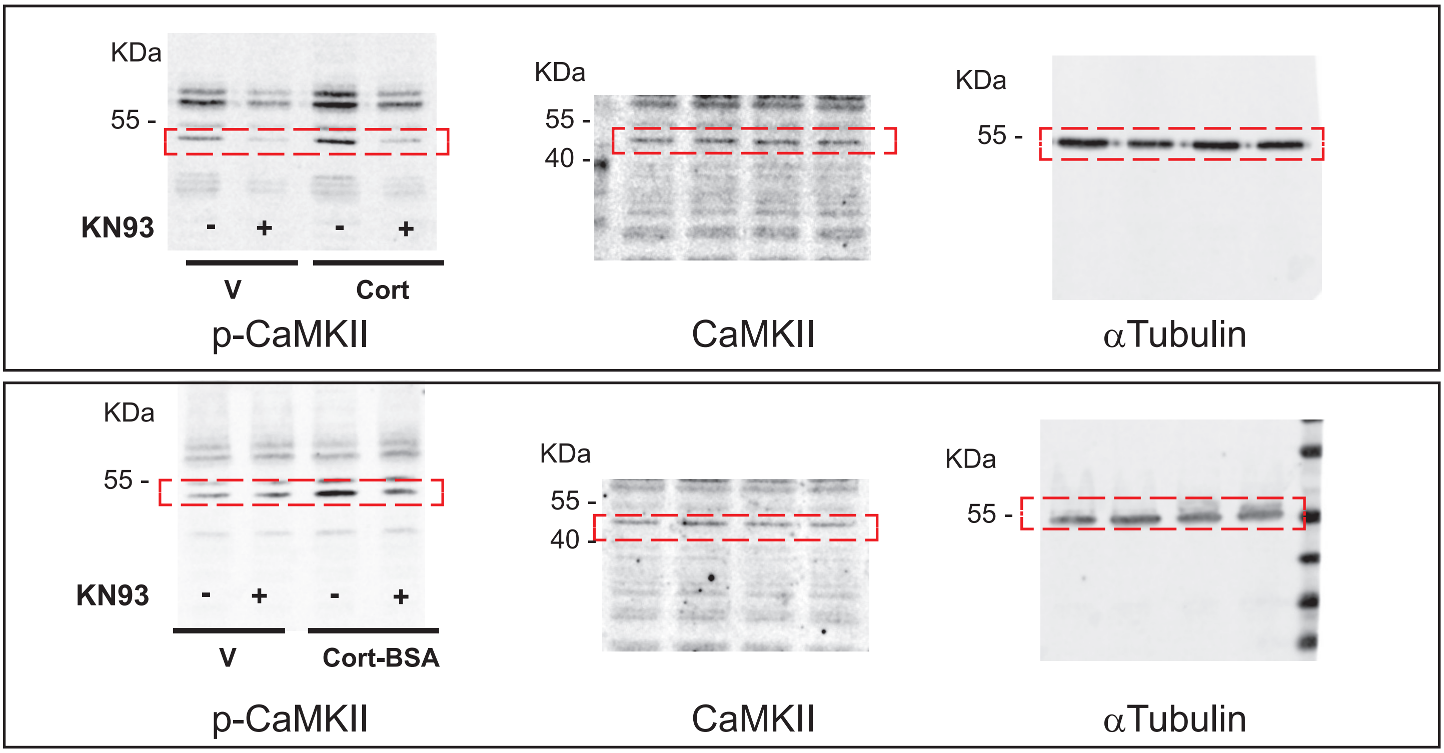

Supplementary Figure S4 b

Figure 1f

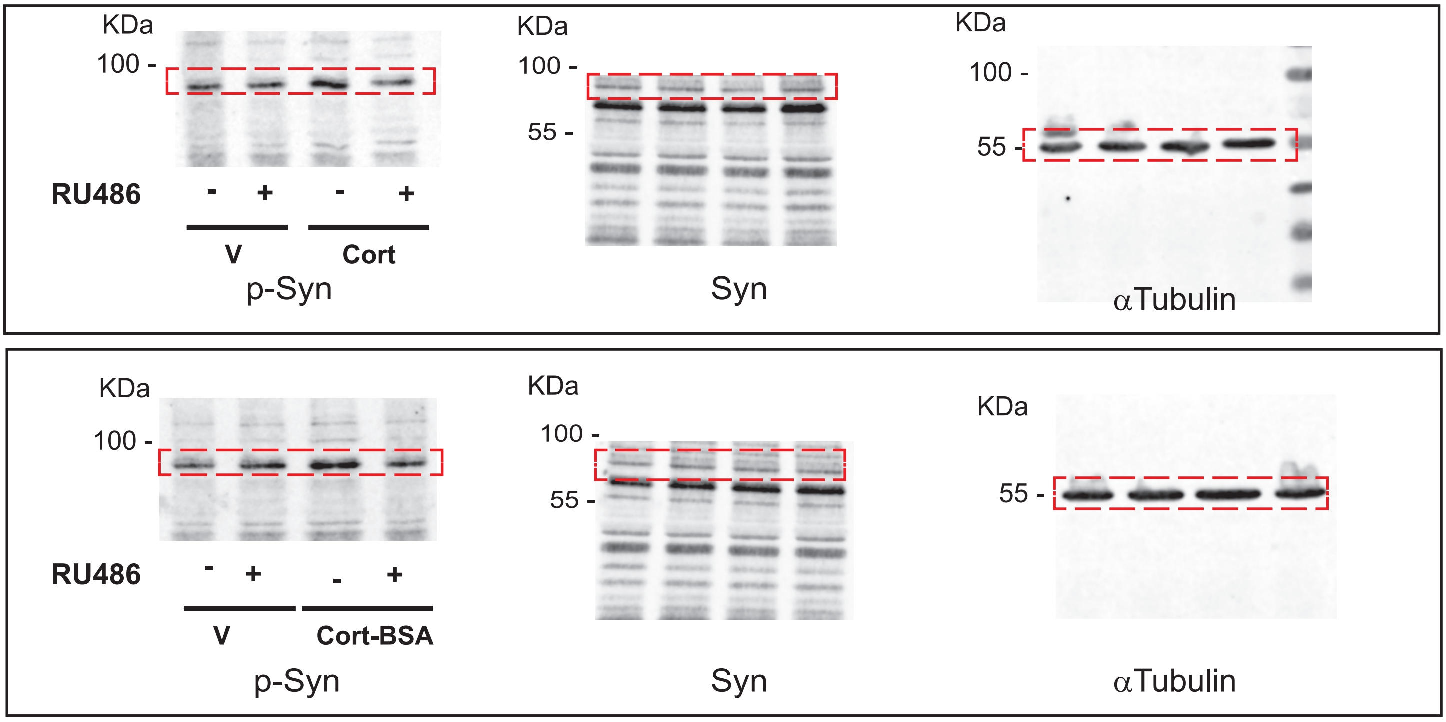

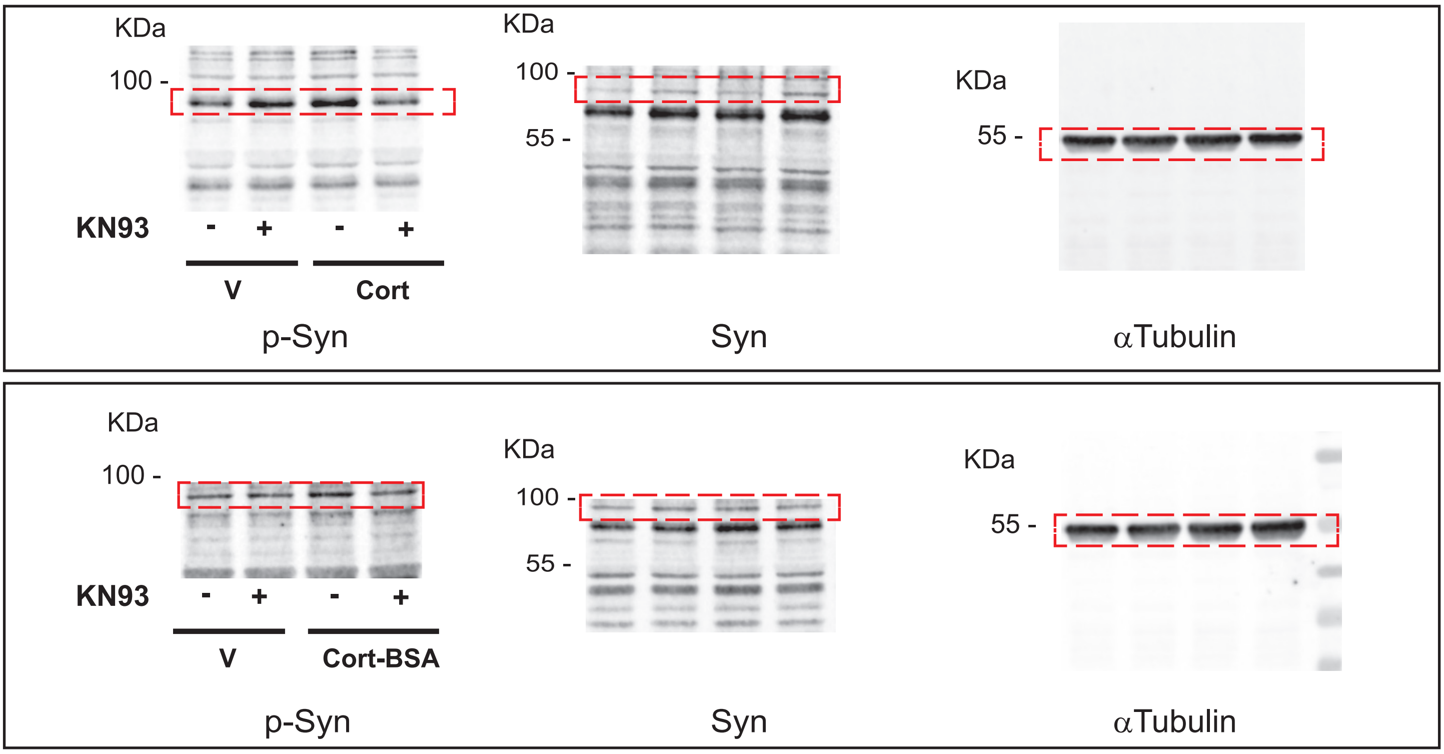

Supplementary Figure S4 c

Figure 2 (upper panels)

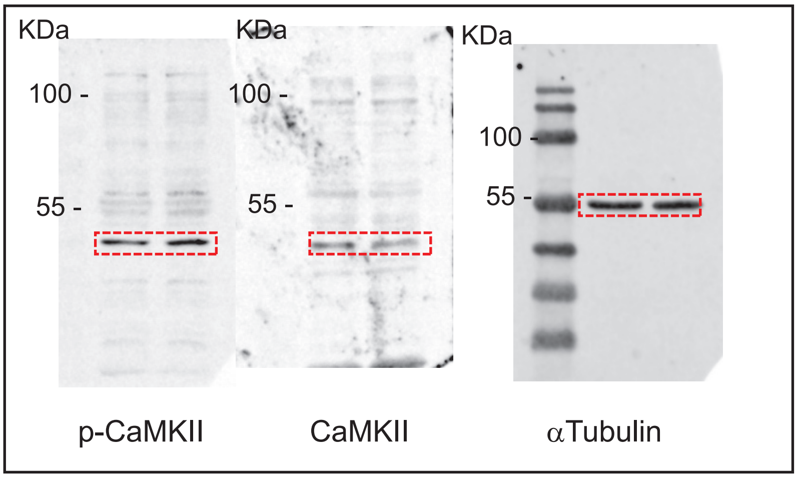

Figure 2 (lower panels)

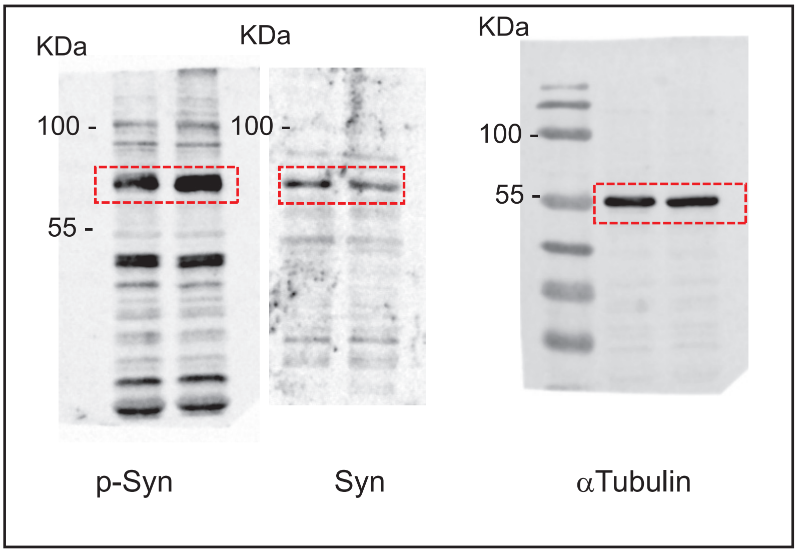

Figure 3a

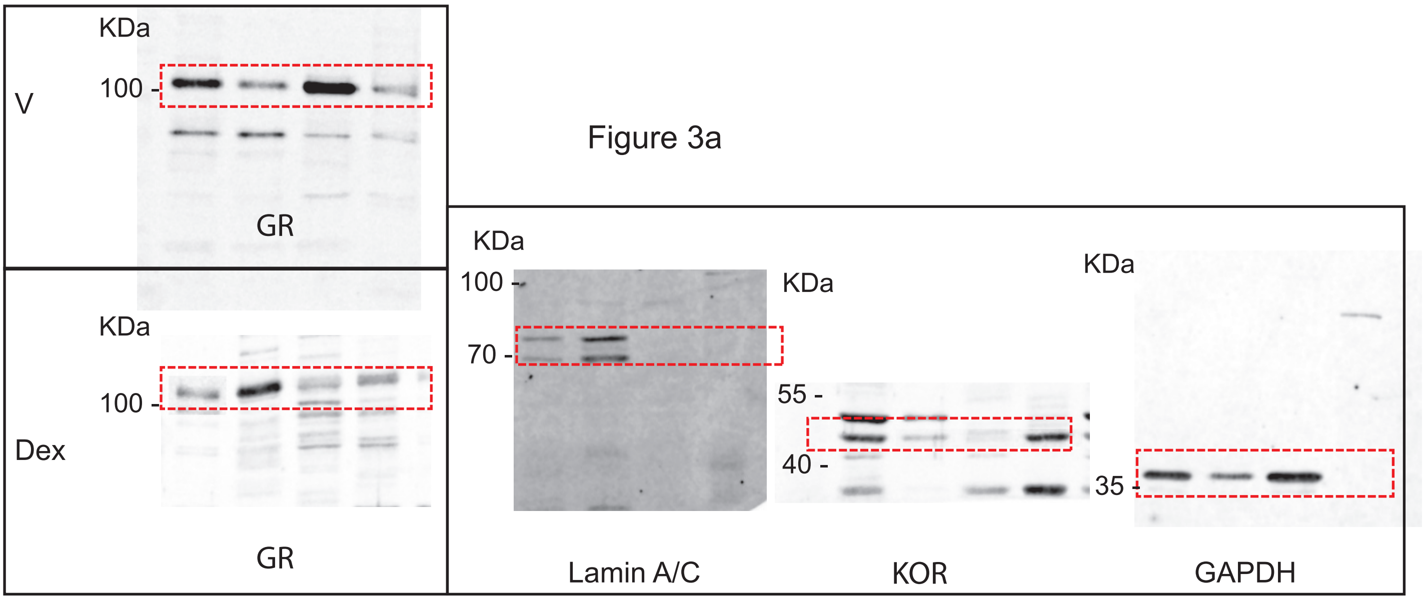

Supplementary Figure S4 d

Figure 3c (upper panel)

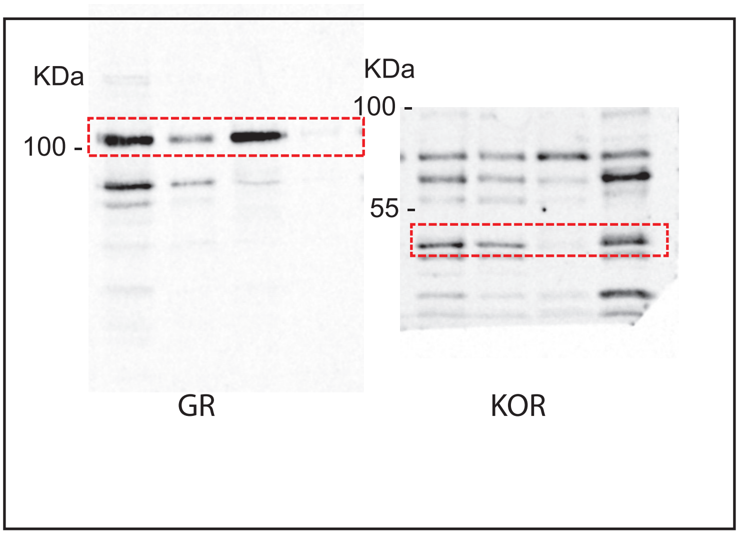

Figure 3c (lower panel)

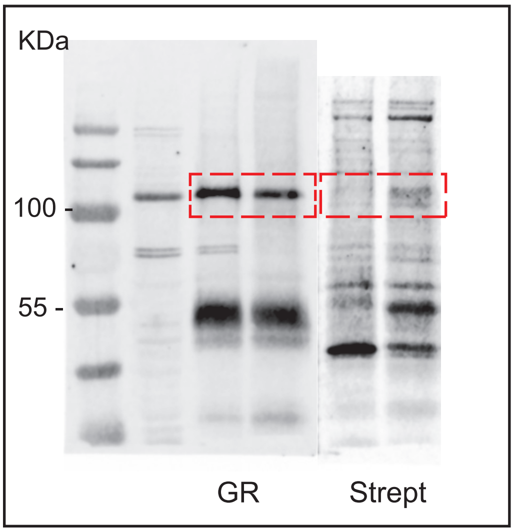

Figure 3d

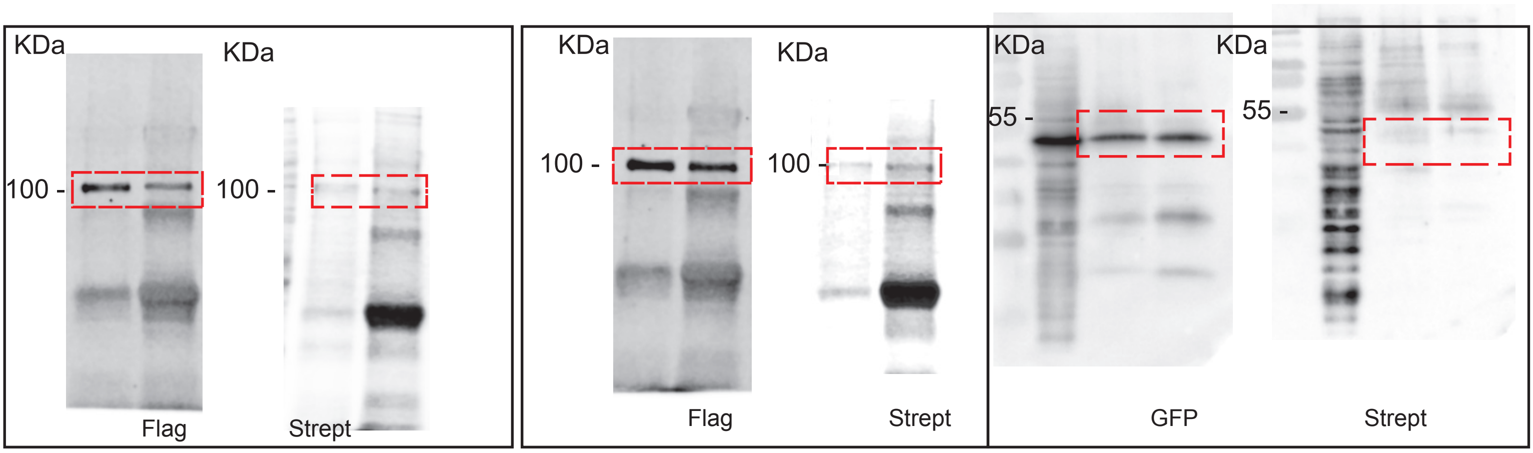

GR-Wt **GR-C/A** **St1**

Figure 4

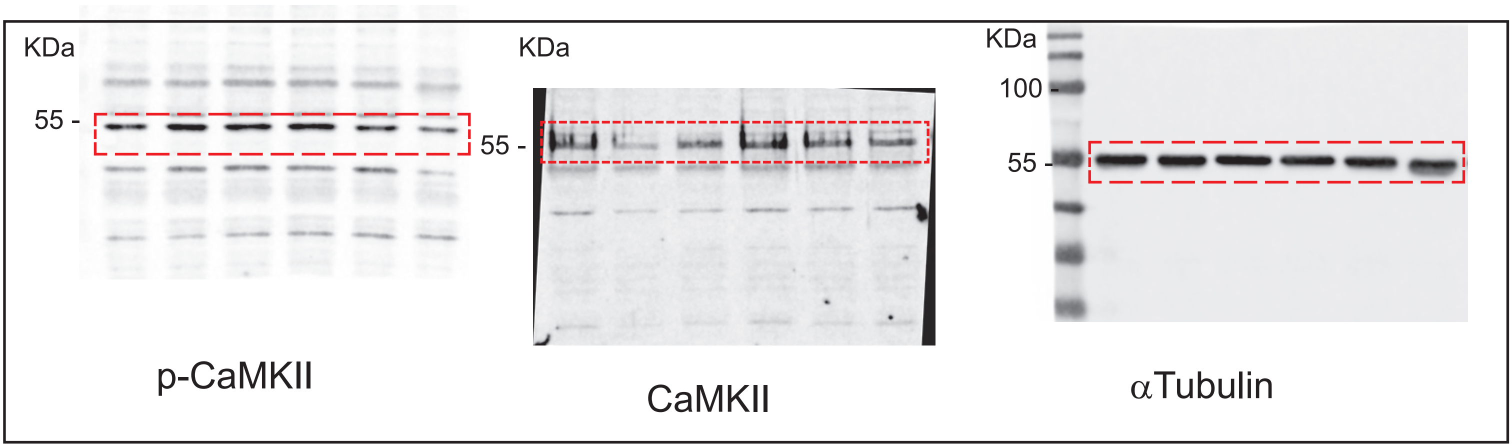

Supplementary Figure S4 e
